# A History of the Break-Bone Fever as It Prevailed in the City of Montgomery, Alabama

**Published:** 1867-04

**Authors:** R. Fraser Michel


### A HISTORY OF THE BREAK-BONE FEVER AS IT PREVAILED IN THE CITY OF MONTGOMERY, ALABAMA, DURING THE SUMMER OF 1866.

By R. FRASER MICHEL, M.D.

Read before the Medical and Surgical Society of Montgomery, January 7th, 1867.

The city of Montgomery was visited during the past summer by an epidemic of break-bone fever, which taxed the energies of the profession to their utmost extent.

The reader will see, as we progress in the narrative, that we have correctly named the disease. Many of the most prominent symptoms characterizing the summer and autumunal fever of the West India Islands, in 1827, were absent, as well as those marking the epidemic which prevailed in the southern sections of the United States in 1828.

Prof. Dickson, from whose able pen we derive much that we know of dengue, is so clear in his symptomatology of this disease, that we incline to the opinion that the epidemic we have just passed through was not one of dengue, but rather resembled what Dr. Rush has described as having occurred in Philadelphia in 1780, and Drs. Dickson, Wragg, Happoldt, and Arnold, as prevailing in Charleston and Savannah, in 1850.

We concur fully in the opinion expressed by Dr. C. Happoldt in his inaugural dissertation, published in the *Charleston Medical Journal*, for 1851, when he says:—"If the essential and characteristic symptoms of dengue be, a fever of nearly uniform duration, preceded by arthritic inflammation, and succeeded, after an interval, generally regular, by a secondary fever, which is almost invariably attended by a uniform eruption, and followed by violent pains of a rheumatic character, must it not be entirely distinct from a form of disease in which these symptoms are either *absent*, or their order of succession entirely different."

The first case of break-bone fever in Montgomery, occurred on the 7th of September, 1866, in the practice of Dr. J., in the person of Mr. M. G., who had not left the city for some months prior to his attack. I was called on the morning of the 8th to see two cases; one, a captain of a steamboat, running between Mobile and Montgomery; the other, a lady, just returned from the Springs, near Macon, Georgia, where she had been spending the summer. Here we see, that, at the outset, three persons were attacked, almost simultaneously, in the eastern, northern, and middle part of our city; and in ten days one hundred or more cases were under treatment. A sudden invasion followed by such rapid development, that any attempt to trace the disease from patient to patient must necessarily end in failure.

The last case we know of, terminated about the middle of November. The epidemic lasted seventy days, being at its height about the middle of October. There was hardly a family exempt; many in which not a member, white or black, escaped;—not a house, from those crowning the beautiful hills which encircle our city, to the banks of the Alabama, but was visited by this searching malady.

I may mention here, that prior to the occurrence of the epidemic, some of the physicians observed a few cases of scarlatina simplex; these cases were easily managed, and occurred during the months of May and June. In July and August, we had intermittent and bilious remittent fever, the type of the latter unusually severe. It is a noteworthy coincidence that in 1850, the same thing was observed to have occurred in Charleston and Savannah; and, as in 1850, Asiatic cholera again visited the North and West, as the cities of New York, Cincinnati, and St. Louis can testify.

Most of the cases began with fever, without previous *malaise* or chill, accompanied by severe pain in the forehead and small of the back, hot dry skin, smoky eye, full but not very frequent pulse; tongue often clean, occasionally red, in most of the cases covered with a yellowish-brown fur, without thirst. This condition usually lasted about eight hours, when the patient would complain of violent muscular pains, more frequent in the lower than upper extremities, followed by intense nausea and vomiting, lasting about 12 hours; the pulse falling gradually to 50 or 60 beats in a minute. Profuse perspiration would then break out over the entire surface, a slight eruption make its appearance, the gastric disturbance disappear, the pains gradually subside, and the patient, though weak, be able to resume his usual avocations; frequently returning to his accustomed place at the desk or behind the counter, forty-eight hours after the invasion of the disease; many, indeed, recovering before a physician could be obtained.

But while this description is a true history of most of the cases (in the proportion, at least, of six out of ten), we observed others of a severer character.

These graver cases of break-bone were preceded by *malaise* for two or three days, followed by chill more or less marked. And here we would call attention to the fact, that where the patient had passed through one or more attacks of malarial fever during the summer, he would not have simple rigor, but a severe chill, lasting in some instances at least an hour; then fever would supervene with its usual symptoms—pulse full, abrupt, rarely over one hundred to the minute; skin hot, dry, with a reddish hue, indicating tendency to capillary congestion; the heat so pungent, indeed, as to burn the hand when laid upon it. Eyes suffused and watery, pain upon moving these organs, especially when rolling them upwards, or when pressure was made upon them; sometimes intolerance of light was a prominent symptom.

Pain in the head, frequently supra-orbital, though in some of the cases pain of a darting kind was experienced; and as Dr. Wragg has described as existing in some of his cases in 1850, "so severe and darting that the patients said they felt as if a sharp instrument was thrust through the brain," and terminating in many cases in delirium. Pains in the limbs, particularly in the lower extremities; not confined, however, to the joints, but rather extending to the muscles. These pains were so severe as to prevent sleep, and several of our patients enjoyed no rest the first two nights of their attack.

The pain in the head produced great uneasiness in the region of the ear; and in certain cases a neuralgic affection of the fifth pair of nerves, was a characteristic symptom, giving rise to severe tic douloureux, as well as earache, which was attended in some cases by slight deafness.

The abdominal pains which we witnessed as occurring in the

Charleston epidemic in 1850, and which very frequently induced us to believe that we had a case of gastritis or enteritis to deal with, were entirely absent in this epidemic, so far as we are able to discover; and the dyspnœa produced by intercostal pain, which was observed as an almost constant attendant of break-bone, in Charleston, in 1850, was never seen during the past summer. The tongue, which at first, was natural, soon became covered with a greyish-brown coating; and the foul, disgusting taste in the mouth, lasting, in many cases, a week after convalesence was established, beggars description—"Oh! what a taste I have in my mouth!" was a constant exclamation. This symptom was, in some cases, attended by profuse salivation to such an extent as to cause some of our patients obstinately to insist that we had given mercurial preparations in the course of our treatment. The continual spitting reminded us very much of the Minorca fever of 1733 and 1748, as described by Cleghorn; which, by the way, Dr. Rush regarded as similar to his epidemic fever of 1780 (break-bone). In one instance (Dr. O.) we are convinced at least a gallon of salvia was secreted in twenty-four hours. Much nausea and gagging occurring in all of these cases, and violent stomatitis sometimes supervening; the abrasion of the buccal mucous membrane in some instances being very extensive, requiring prompt local treatment. We are particular in calling attention to this symptom (sore mouth), as in the epidemic in Charleston, in 1850, it was almost entirely absent.

The sense of taste being so much deranged, we are not surprised to learn from some of our patients that odors were not recognizable, and in others that certain odors predominated; for instance, one of our female patients told us that everything smelt like the muskmelon or cantelope. Her idiosyncrasy with regard to that delicious fruit was peculiar; she detested it extremely, and could detect one if concealed in any part of the house. The nausea, in almost every instance, was attended by profuse bilious vomiting, in many cases, very persistent, lasting from the chill until convalescence.

What we have so far described may be regarded as the first *stadium* of the disease; in some cases this embraced a period of from six to twelve hours, in others from one to three days. We saw no case during the epidemic in which this stage lasted more than three days; while we know that in 1850, we would frequently see it cover a space of five. The fever now subsiding, a perspiration would be observed covering the entire surface; sleep soon supervened of a refreshing character, but attended or followed by an eruption which would usually cover the face and forehead, extending, in some instances, over the entire surface, and with it a feeling of formication, burning in the palms of the hands and soles of the feet. The perspiration, just alluded to, emitted a very disagreeable odor in some few cases. A professional friend informs us that one of his patients insisted upon continual change of bed-linen, the odor, which he pronounced "intolerable," he described as of a strongly urinous character.

The eruption varied in almost every case, resembling, in many instances, that of scarlet fever, and accompanied with a slight anginose embarrassment. Then again an erysipelatous blush would suffuse the face and hands; so persistent in some cases as to excite the belief that desquamation must result; but passing off slowly during convalescence, leaving the cuticle uninjured. In other cases, a lichenoid or miliary eruption, resembling prickly-heat or nettle-rash, with intense itching, would come out; and when this condition obtained in the young subject, desquamation was sometimes extensive, affecting the tissues beneath the cuticle. A professional friend informs us that in one of his cases, his little patient lost the nails of the toes and fingers. We saw no cases resembling rubeola, or impetigo, no petechial, variolous, phlegmonoid, purpurous, or papular eruption. And while referring to petechiæ, let us say we did not witness a case of hæmorrhage (as we did in 1850), from the gums, fauces, stomach, or bowels; nor did I hear of any during the epidemic, except a few cases of epistaxis.

These eruptions occurred during the second stage in most of the cases; but they would occasionally make their appearance during the febrile paroxysm, and extend even into convalescence. They had no special predilection for any particular part of the body, sometimes appearing on the forehead, face, and upper part of the chest, but just as often on the extremities or abdomen. In some instances there was no eruption to be observed, but towards the latter part of the epidemic there was hardly a case that did not present it in a well marked degree.

Our attention was particularly directed to the eruption in the negro cases, from what Dr. Dickson observed with reference to this class; and we endorse the fact, that very few negroes suffered in this way. We attended twenty-three cases among the blacks, and only two presented an eruption. The erysipelatous blush alluded to above, in some instances covered the hands entirely, causing them to become extremely sensitive, so much so as to entirely prevent their use as prehensile organs. When there was any desquamation, of course the itching of the skin was present; in some cases severe and intolerable, and attended with swelling of the face.

The convalescence was slow, lasting a week or ten days, with anorexia. We saw no case during this epidemic which reminded us of the extremely slow convalescence of some of our cases of break-bone fever in Charleston, in 1850. It was not unusual then for a patient to take three weeks or a month to convalesce. Nor did any case under our care end in typhoid disease. There was no tottering or irregular locomotion; no staggering, uncertain walk, requiring the use of a crutch or stick, as we often see in dengue; no stiff affected gait, nothing peculiar. When you met a friend whose absence from his accustomed occupation had been observed, you could not tell by any external sign that he had had break-bone fever; you would only ascertain the fact by making inquiry to that effect.

And, now, the question naturally suggests itself, were there any cases of relapse? Very few, we answer, if any. We heard of none, at least, and would invite attention in passing, to the fact, that this was a common occurrence in the epidemic of break-bone fever in 1850. Dr. Wragg, in his able essay on this subject, says: "Another remarkable feature of this fever was the tendency to relapse, or the ease with which it was brought back by premature exercise, fatigue, or any kind of exposure. We have seen it recur under these circumstances, as often as three times, and on each return, with marked aggravation of all the symptoms. Often, when the first attack had been exceedingly mild, the subsequent ones would be excessively severe, and always of longer duration than the first." Here there is a marked difference between the two epidemics of 1850 and 1866; we, however, believe the diseases were the same, though in some features they may have differed.

As during epidemics of Asiatic cholera, there are cases (if they can be called such) in which slight pain is felt in the abdomen, and even considerable borborigmus is apparent without any other symptoms characterizing this disease; we saw many patients, who, with no fever, had some one or more of the signs of the epidemic disease, as vertigo, nausea, syncope, deafness, pain in the head, back, and limbs, followed by lassitude, *the eruption not appearing*. We must call these cases *break-bone fever without the fever*, and we incline to the opinion that these symptoms were sufficient to give those who so suffered, immunity from the severer form of the disease during the epidemic. Persons of all ages were subject to the disease—the child at the breast, and the venerable elder of four score years, both male and female, white and black; and it created a surprise to see the aged recover so promptly from the effects of the malady; indeed, an old professional friend remarked, "that he was going down the hill very fast before he was attacked, but since he had recovered he felt better than he had been for years, and he believed he was a stronger man."

Some of the sequelæ in the Charleston epidemic of 1850 were absent in this—for instance—the boils, carabuncles, and abscesses were not found. The enlarged and deformed joints with extensive effusion were rarely seen. The remarkable loss of hair was not present in this epidemic, and even during the attack some of the symptoms of the Charleston break-bone were slight or totally wanting — for instance — the intense nervous trembling, which marked the onset of the disease, the jerking and twitching during the attack were not witnessed; and, though *one* case of the disease was ushered in with convulsions, no death from this complication was recorded. We heard of no pregnant women aborting, as in dengue, from the suffering and pain induced by the fever, and though, as we have already noticed, audition was much impaired, no case of permanent deafness was observed. During the progress of this disease, we saw no case developing those hallucinations, resembling the demons of *mania-a-potu*, so painfully common in 1850. On the other hand, we would invite attention to the extreme debility and exhaustion in which our patients were left, resembling the epidemic just alluded to, and in some cases, when contrasted with the duration and want of intensity of the malady, truly surprising. In many cases the patient was even threatened with syncope, upon suddenly changing from the horizontal to the erect position, showing the enfeebled condition of the heart.

*Pavi passu* with the epidemic, we observed many cases of ophthalmia, some occurring among our break-bone patients, and appearing as a prominent symptom. Most of the cases engrafted upon the epidemic disease were quite severe and stubborn in character—the lids extremely grandulated, and in some of the severer cases, corneitis, sclerotitis, and even iritis, with great photophobia, were observed.

Upon the causes of the epidemic we will have little to say. The atmospheric changes, prior to, and during the epidemic, may lead those more conversant with meteorology to form some opinion, however, and we will, therefore, give as accurate an account of them as the limit of this paper will permit.

The spring was peculiarly cold, much more so than is usually observed in this latitude, and the cool weather continued into June. We had, during this season, more rain than usual, and the winds were from the south-west almost exclusively. After the middle of July, the weather became hot, and continued *very hot* until the middle of September. An old and observant citizen tells us that it was hotter in Montgomery during that period than he had ever felt it before, and he resided in the place forty-seven years. The thermometer ranged in mid-day between 90° and 95°. He called our attention to another fact in connection with this heat, worthy of notice. "That during that time we had less thunder and lightning than he had ever known before;" an indication of the peculiar electrical condition of the atmosphere. The nights were oppressively hot, the thermometer, in the early part of the night, registering usually from 85° to 87° F. During this time, that is from the middle of July to the middle of September, the winds were westerly and easterly alternately. At this time the drought was excessive and destructive to vegatation, as the scanty crops demonstrate; and, singular to relate, from the latter part of August to the middle of October, during certain spells, that is, when the wind would suddenly shift from west to east, heavy falls of rain ensued, deluging the country. Slight frost was observed in and around the city on the 5th of November, but not severe enough to destroy vegetation, and it was not until the night of the 21st of November, that the cold was intense enough to kill garden plants. Here our epidemic differed from dengue. It did not occur in a pleasant, cool, and temperate season, nor did it select the winter or early spring, as is its wont, nor were any foggy mornings to be observed.

Whilst treating of the causes of this disease, it is, perhaps, proper to add that we had an epidemic of small pox, beginning in the spring of 1865, and ending in the spring of 1866. This, associated with what we have already mentioned, "that several cases of scarlatina simplex occurred during May and June." would lead us to ask, what impression this condition of things could make upon the atmosphere of Montgomery? It appears to us that it would be very rational to believe, that if we had a *fever*, the skin would play a very prominent and important part in its history.

At this point, we would request the reader to examine the character of the summer and fall seasons in Charleston, in 1850, so clearly and accurately recorded by Dr. W. T. Wragg, in his paper on Break-bone Fever, as well as the same seasons in Philadelphia, in 1780, described by Dr. Rush, and in Montgomery, in 1866, as now furnished by us; and I think a striking parallel will be observed, or, to say the least, a very singular coincidence.

During the epidemic, the usual intermittent and remittent fevers continued; they were more extensive in their range, however, covering a much larger surface than ever before. Certain localities, which hitherto had been regarded as healthy, and which were used as summer resorts by the citizens of Montgomery, were quite sickly this summer, and persons did not know where to take refuge to insure health. This condition of things (an epidemic of break-bone fever, accompanied by intermittent and remittent fever) was very embarrassing, and the physician could not be too cautious in pronouncing dogmatically upon the various cases which marshalled themselves before him for treatment. We saw many cases of the epidemic fever remitting and even intermitting.

We now come to the most important part of our discussion—the pathology of the disease. This is always a difficult question to determine with regard to break-bone fever; the fact, that no deaths occurred, and that consequently no *post mortem* examinations were made, is one reason of its obscurity. *No death occurred from this disease during the epidemic*, and nearly half the population were afflicted with it.

We incline to the opinion already expressed by others, that the disease is located in the nervous system, and that, secondarily, the cutaneous surface becomes affected—*a nervo-cutaneous* affection. That the nervous system is first affected I have no doubt: observe the pain, tremor, rigor, delirium, and other phenomena already described; then the cutaneous system, as is demonstrated, first, by the feeble capillary circulation, the reddened surface, the eruptions, the desquamation, and the intolerable itching, almost always observed during convalesence.

How any one can confound such a disease with yellow fever, when we consider the uræmic poisoning, the terrific hemorrhages from eyes, gums, stomach, and intestines and ovaries, which occur in the latter; or, on the other hand, with bilious remittent, so well characterized by its *post mortem* appearances, the inflamed gastro-intestinal mucous membrane, the enlarged liver and engorged spleen, seems to us matter of surprise. No! Break-bone fever is a peculiar affection, and one which deserves more notice than it has hitherto attracted.

The epidemic may have been taken for dengue, and to this point we would invite special attention. Prof. Samuel Henry

Dickson, thus describes dengue, page 606, vol. II: "Dengue usually made its invasion with pain, stiffness, and swelling of some of the smaller joints, often of the muscles of a limb, rigidity of the neck, aching of the back and loins. These pains were followed, after an uncertain, though generally brief period, by headache, suffusion of the eyes; abrupt, full, frequent, pulse; hot, pungent, dry skin, restlessness, thirst, and other tokens of febrile excitement. The fever did not remit, but declined and disappeared in a great majority of cases on the second or third day. In this early stage the tongue was generally clean, and the stomach quiet; but sometimes there was nausea or even vomiting, the determination to the head was occasionally violent. Instances occurred in which delirium was among the first symptoms, coming on at the commencement and enduring until the subsidence of the febrile paroxysm. At this time the skin lost its heat and dryness, becoming relaxed, with abundant perspiration, and the local pains were all lessened in degree. A sort of miliary eruption, or rash in some persons, attended this sweating stage, and in a few others preceded both the local pains and the fever. It was, however, as connected with this first stage of dengue, a very inconstant symptom, seeming rather a coincidence than a symptom. The pains of the joints and muscles which, as has been said, were diminished in severity at the subsidence of the febrile exacerbation, did not go off entirely; a degree of swelling, stiffness, and tenderness of the affected parts remaining permanently, though varying much in intensity in different individuals. This condition of things constituted a sort of deceptive interval between what may be described as the first and second stages of this strange disease. Many now believed themselves to have passed through the attack, and attempted to resume their ordinary occupations; but soon had occasion to discover that their sufferings were by no means at an end. On the third or fourth day, there being no fever present, or a very obscure degree of it, the tongue would begin to be coated with a yellowish fur, and the stomach would become distressed, uneasy, and irritable. The patient was now low-spirited, fretful, and anxious. Vomiting come on in some, with great languor, lassitude, debility, and restlessness at night. This was regarded as the most oppressive and insufferable of the stages of the malady. On the fifth or sixth day from the invasion, the period varying somewhat in different individuals, the annoying symptoms just described were relieved by the coming out of an abundant eruption, met with so constantly and in so very great proportion of the cases, that it clearly demands to be considered a characteristic and essential circumstance in the history of the disease. It consisted of minute papulæ, somewhat elevated, of a florid red, and distributed in irregularly shaped patches; the feet and hands being somewhat swollen, with a sense of numbness and thickening. It appeared first on the face, then on the trunk and thighs, gradually spreading to the extremities. It resembled scarlatina more than measles, in the hue and aspect of the skin, but was less diffused or confluent than either. When fully developed, it was attended with some itching and burning of the surface, and at this time a second febrile paroxysm came on, with return or aggravation of the muscular and arthritic pains. Inflammation and enlargement of the lymphatic glands in the neck, axilla, and groin, attended in a good many cases; these parts being apt to continue swollen and painful for some time after convalesence was finally established. In a few instances suppuration of these tumors took place. The eruption disappeared after two or three days' duration, becoming gradually paler, with some desquamation of the cuticle. Of all the symptoms of dengue the affection of the joints was the most tenacious and troublesome, adhering for weeks to some patients, and constituting a sort of permanent lameness or loss of mobility. Nay, even so late as 1835, some of the population of cities visited by this plague, persisted in speaking of the rheumatic or quasi-rheumatic decrepitude and pain under which they labored, as the effects of dengue.

"Pregnant women, when attacked, were very liable to abortion, and a remarkable number of miscarriages and premature labors occurred. A sore mouth was among the frequent symptoms, ulcers formed in the mouth. Dengue is to be classed properly among the exanthemata. It is an eruptive fever of distinct and specific character. Its essential symptoms are, in the first stage, a painful affection of the joints and muscles; and, in the second, divided by an interval obvious and sufficiently regular, a cutaneous eruption. The arthritic inflammation of the first stages was attended by fever of the ordinary inflammatory type, of twenty-four to forty-eight hours' duration. The eruption was preceded, as is usual in the exanthemata, by considerable gastric oppression, with nausea, and sometimes vomiting."

We must regard the epidemic we have described as differing in many respects from the dengue, so beautifully portrayed by that accomplished scholar and physician, Prof. Dickson.

Look carefully at the premonitory symptoms, the kind and severity of the pains which marked the onset, continuance, and termination of the two diseases. Observe the intermission, not accidental, not occurring by chance, but regular, never deviating, always coming to the relief of the sufferer, and to be counted with certainity by the hands of the clock. Then examine the secondary fever, accompanied always by an eruption "that clearly demands to be considered a characteristic and essential circumstance in the history of the disease." Look at the prompt convalescence of dengue, with the deformed and enlarged joints, the patient staggering about, bald-headed, with stick in one hand, or crutch in both—remark the common accident of abortions and premature labors, and then say if this condition of things resembles, however remotely, the epidemic we have just passed through.

We believe that the fever described by Dickson, Wragg, Happoldt, and Arnold, in 1850, is the disease we had to contend against this summer in Montgomery; beginning in the nervous and cutaneous system, and not in the ligamentous and fibrous.

It may not be irrelevant to present at this point the description of the epidemic fever which occurred in Philadelphia, in 1780, given by Dr. Rush: "It affected all ages and both sexes. Medical men would seem to have been specially liable to it. No other febrile disease was observed during its prevalence. It came on sometimes with rigor, seldom with a chill. Many instances occurred in which it was introduced by delirium. The pains which accompanied it were excessively severe in the head, back, and limbs; in some they affected the neck and arms, and in one case produced a difficulty of moving the fingers of the right hand. Hence the disease was sometimes believed to be a rheumatism, but its more general name among all classes of people was the break-bone fever. A nausea universally, and in certain instances vomiting attended. The pulse was full and quick, but never hard; there was little or no thirst. A rash often appeared on the third or fourth day, accompanied by a burning in the palms of the hands and soles of the feet. Convalescence was slow and tedious. The disease was seldom fatal. The treatment required was singularly mild." Now, we see in this description of Dr. Rush, an almost accurate account of the fever we witnessed in the city of Montgomery, in 1866, and in Charleston, in 1850.

Frost (as it always has done) terminated the epidemic. After the middle of November we heard of no new case. No stranger coming into the atmosphere after frost, *contracted* the disease, although those who had spent the summer here were liable to an attack a few days after this truly welcome visitor.

We do think it contagious;—many cases found their way to the different villages and towns in the neighborhood, by railroad and stage, or slowly along the beautiful waters of the Alabama; but we heard no voice echoing its painful though harmless presence as epidemic in any of the garden spots of the State.

The period of incubation varied from five days to a week; though we knew of one patient who was attacked forty-eight hours after his arrival in Montgomery.

The prognosis was, of course, favorable, for when a disease never destroys life, or leaves any organ of the body seriously damaged, we are compelled, no matter how painful or seemingly severe it may be, to pronounce it harmless.

We would remark, in this connection, that although cases of bilious remittent, some of them congestive, occurred during the summer, we heard of no case of break-bone assuming the congestive type; a fact worthy of special notice.

With regard to the treatment of this disease, there is of course little to be said; almost all the physicians here treated it according to the *medecine expectant.*

If we arrived in time to find our patient with chill, warm stimulating applications were made to the spine and extremities; we preferred the spirits of turpentine. A warm foot-bath was an excellent adjuvant, and with a few blankets thrown over the patient, we awaited the cessation of this symptom. During the fever which followed, we did no more than attempt to relieve the troublesome symptoms as they arose. If our patient was costive, the bowels were evacuated with an enema, sometimes by laxatives, a sedlitz powder, or a dose of castor oil. In this stage refrigerants were freely used; cool iced lemonade, and sponging the entire surface of the body with vinegar and water; cold applications to the head, as by shower-bath and ice in bladders to the frontal surface; frequently pepper plasters to the temples gave relief. The nausea and vomiting was quited by sinapisms over the epigastric region, and by the internal administration of magnesia or lime-water. When the fever was subsiding, a warm infusion of boneset was ordered; or, what our patients preferred, hot lemonade. In some of the cases where an intermission in the febrile paroxysm was observed, the administration of fifteen grains of the sulphate of quinine in divided doses seemed to control the disease, and to hasten convalesence.

In those cases in which the throat was affected, a gargle of the chlorate of potash was advised, or our patient permitted to dissolve in the mouth every hour, until relieved, the chlorate of potash lozenger prepared by Hegeman & Co. The detestable taste in the mouth, which persisted even after convalescence, is difficult of relief. We found that claret wine, freely administered, gave more speedy relief than anything else. The administration of opium lessened the muscular pain, promoted the action of the skin, and induced sleep. To relieve the neuralgic pains, the sulphate of morphia injected hypodermically, proved eminently successful. The application of olive oil and lime water, as well as alkaline washes to the skin, were found the best agents to relieve the burning and itching of that sentient surface.

The epidemic was certainly a mild one, when compared to that we witnessed in Charleston, South Carolina, in 1850; but in all its essential features, it resembled the latter so closely, that we think we must, without hesitation, class it with that form of epidemic fevers which so far seem exclusively confined to this Continent. The English and French writers say nothing of it. As a disease generally recognized, or of wide-spread, or even occasional occurrence, it seems to be unknown in Europe. We have therefore thought it not unwise to put the account of this epidemic on record, if only for the sake of the future medical historian.—*Southern Journal of Medical Sciences.*

